# Editorial: Insights in Alzheimer's disease and related dementias: 2022

**DOI:** 10.3389/fnagi.2023.1279870

**Published:** 2023-09-22

**Authors:** Agustín Ibáñez, Allison B. Reiss, Nilton Custodio, Federica Agosta

**Affiliations:** ^1^Latin American Brain Health Institute (BrainLat), Universidad Adolfo Ibáñez, Santiago de Chile, Chile; ^2^Cognitive Neuroscience Center (CNC), Universidad de San Andrés and CONICET, Buenos Aires, Argentina; ^3^Global Brain Health Institute (GBHI), University of California San Francisco (UCSF), San Francisco, CA, United States; ^4^Trinity College Dublin (TCD), Dublin, Ireland; ^5^Department of Medicine and Biomedical Research Institute, NYU Grossman Long Island School of Medicine, Mineola, NY, United States; ^6^Department of Neurology, Instituto Peruano de Neurociencias, Lima, Peru; ^7^Unit of Diagnosis of Cognitive Impairment and Dementia Prevention, Instituto Peruano de Neurociencias, Lima, Peru; ^8^Vita-Salute San Raffaele University Milan, Milan, Italy

**Keywords:** Alzheimer's disease (AD), dementia, brain health, neurodegeneration, animal studies, human studies

## Introduction

Dementia impacts over 55 million individuals globally, creating a pressing public health concern characterized by cognitive and functional decline beyond typical aging. As highlighted by the World Alzheimer Report (Alzheimer's Disease International, 2022) and the World Health Organization (2022), Alzheimer's disease (AD) and related dementia (ADRD) are the principal cause of disability, dependence, and death among the elderly. Its repercussions, both personally and socially, vary in magnitude, often reflecting the inequality gradients of the respective nations. Today's research in aging, dementia, and other brain diseases is moving away from one-size-fits-all models to embrace multifaceted, heterogeneity-aware, and diversity-driven paradigms (Alladi and Hachinski, 2018; Parra et al., 2018, 2021, 2022; Dehghani et al., 2021; Deco et al., 2022; Frisoni et al., 2022; Ibanez, 2022; Moguilner et al., 2022; Ibanez and Zimmer, 2023; Ibanez et al., 2023; Maito et al., 2023; Santamaria-Garcia et al., 2023). This dynamic evolution in dementia studies is pioneering new interdisciplinary connections, spanning various methods, scales, populations, and regions. It dives into genetic diversity, protein aggregation patterns, synaptic activities, disparities across multiple fronts, advanced biomarkers, and the interplay of environmental triggers with physiological stress mechanisms. Moreover, a trend toward non-generalized samples and unique research designs in neuroscience has become evident. Years of research have enhanced our understanding of AD's underlying mechanisms. Technological and diagnostic advancements have paved the way for therapeutic innovations, as evidenced by the recent FDA approval of drugs such as Aduhelm and Lecanemab, marking progress in the field. While research in ADRD has significantly broadened over the years, these advancements have led to improved patient outcomes. However, challenges persist, such as complexities related to heterogeneity in disease presentation, the lack of early detection biomarkers, and the absence of massive development of disease-modifying therapies.

As we journey into the third decade of the twenty first Century, remarkable strides have been made in Alzheimer's disease and related dementia. Frontiers has curated a series of Research Topics to spotlight these advancements in Aging Neuroscience. This special edition, titled “*Insights in Alzheimer's disease and related dementias*,” offers a concise overview of selected studies to enlighten and guide the research community.

There has been a growing interest in different factors impacting cognitive function, dementia, and Alzheimer's disease (AD). Costa-Laparra et al. delved into the relationship between specific genetic factors, such as the APOE ε4 allele and G206D-PSEN1 mutation, and mitochondrial abnormalities in AD. Their study demonstrated that these genetic variations could lead to mitochondrial fragmentation and an increased propensity for oxidative stress-induced cell death, potentially contributing to AD and neurodegeneration. Yang et al. explored the impact of hearing aids on cognitive function in middle-aged and older adults with hearing loss, including those with AD, dementia, and depressive symptoms. Their meta-analysis revealed that for those with AD or dementia, hearing aids did not significantly enhance cognitive function. Thus, the efficacy of these aids in non-demented subjects remains uncertain.

In a diverse population, Chen et al. devised a predictive model for cognitive impairment in elderly illiterate Chinese women (*n* = 1864), determining that age, daily living activities, and waist-to-height ratio were critical predictors. Meanwhile, Hwangbo et al. drawing longitudinal data from South Korea (*n* = 794,448), identified physical inactivity, diabetes, and hypertension as the top modifiable risk factors for dementia, emphasizing the importance of lifestyle modifications for prevention. Lastly, Balzamino et al. used a Reeler murine model, deficient in the Reelin protein, to show parallels between pathological hallmarks of AD and ocular degeneration, suggesting potential shared pathways in neurodegenerative diseases and age-related macular degeneration.

Collectively, these studies underscore the multifaceted nature of cognitive decline and dementia, hinting at diverse intervention points for prevention and treatment. This Research Topic showcases handpicked studies from Alzheimer's disease and related dementia research. Our aspiration is that these insights not only spotlight past advancements but also illuminate the upcoming challenges in the domain.

## Author contributions

AI: Conceptualization, Funding acquisition, Writing—original draft, Writing—review and editing. AR: Writing—original draft, Writing—review and editing. NC: Writing—original draft, Writing—review and editing. FA: Writing—original draft.
